# Does CSR Activity Amount to Socially Responsible Management?

**DOI:** 10.1007/s40926-020-00158-6

**Published:** 2021-01-28

**Authors:** M. John Foster

**Affiliations:** Kingston Business School, Kingston University, Kingston Upon Thames, KT2 7LB UK

**Keywords:** CSR, Socially responsible management, UK listed companies

## Abstract

In essence firms or companies are usually thought to exist to make products for or provide services of some sort to third parties, other companies or individuals. The *philosophical* question which naturally arises then is ‘to the benefit of whom should a firm’s activities be aimed?’ Possible answers include the owners of the firm, the firm’s employees or wider society, the firm’s local community or their host nation. It is because of firms’ location within a wider society that the issue of corporate social responsibility arises. The issue is do they contribute in a positive way to the fabric of society. In this paper we conduct an exploratory investigation whose research questions, broadly, are whether there is public evidence of corporate social responsibility activity by firms listed in the UK and to what extent, if any, such activities may amount to genuinely socially responsible management by the firms. We examined the most up to date annual reports of a split sample of 36 firms listed in the FTSE 350. The short answers to the two research questions above are: to some degree and no by some margin, based on data from the sample firms.

## Introduction

In essence firms or companies exist to make products for or provide services of some sort to third parties, other companies or individuals. The *philosophical* question which naturally arises then is ‘to the benefit of whom should a firm’s activities be aimed?’ Possible answers include: the owners of the firm; the firm’s employees; the firm’s local community or their host nation.

It is because of that third possibility, and to a degree the second one, that the term corporate social responsibility (CSR) has obtained wide currency in the post-World War II era. Its meaning is obvious from the phrase, but, for the avoidance of doubt, ‘social responsibility’ is that responsibility that an individual or organisation has to the wider society within which they reside. Hence, ‘corporate social responsibility’ is that social responsibility, or duty, which relates to a corporation or firm. (Bowen (1953) provides an early, clear elaboration, if more detail is desired.) If, as the phrase CSR suggests, companies or corporations have social responsibilities, this opens the door to the notion of their having an ethical or moral duty to conduct their business in a socially responsible manner. Not everyone agrees with this proposition, led infamously by Friedman (1962), but the idea that firms do have at least some minimal social responsibility has become increasingly accepted over the 50 plus years since Friedman argued otherwise. Indeed it is well enough established that the concept is recognised by various international bodies such as the UN via its “Global Compact” (UNGC, 2006) and the OECD through its “Guidelines for Multinational Enterprises” (OECD, 2011).

From a perspective of simple logic, it should be possible to so embed this sense of responsibility into the corporate mind and heart that we could have a business which would be socially responsibly managed. A truly socially responsibly managed firm would be organised such that its very being is socially responsible. Hence we use the term SRM here to mean an overall approach to management which fully embraces such a philosophy of social responsibility. We have argued elsewhere, Foster (2018), that it is possible to run a successful, profitable business on a socially responsible basis. Indeed the argument ran that firms can actually build socially responsible practices into their very fabric or, to put it another way, make them part of the foundations on which firms may be built. To support that proposition, case study evidence from seven firms was adduced which featured family firms, some large some small, but it was suggested that the nature of the ownership of the case firms was in itself important. Family ownership, a particular type of close ownership, enables those directing a company to make choices which might otherwise be thought more contentious by shareholders committed to maximising their own wealth. Indeed it may be argued by some to be contrary to the dictates of the UK Companies Act (2006), which gives primacy to the interests of owners or shareholders, whilst allowing the possibility of wider stakeholder interests. While it is true that family firms may be able to act in a more socially responsible fashion than may be the norm, some have argued that they may not necessarily so act, see Cruz et al. (2014), but the choice is at least theirs to make.

CSR is not a synonym for socially responsible management (SRM) but they are certainly closely related and CSR is now a fairly common feature of the public utterances of listed companies. In the world of the listed firm, in the UK and elsewhere, an interesting question then is whether there is public evidence of CSR activity by such firms (i.e. listed firms); and, if such evidence exists for a given firm (if it does at all) then to what extent does that evidence indicate that the firm is following a policy of fully blown, socially responsible management, with socially responsible policies embedded into the total framework of the firm’s activities.

This is the topic we explore in this paper. After discussing the background concepts in a little more detail, we shall look at some empirical evidence from a sample of companies listed on the London Stock Exchange. In order to meet the overall research objective we determined that it would be helpful to break it down into three questions, which were:

- Where within each annual report (AR) did the company report on CSR activity?
- What did they report themselves to be doing on the CSR front and to what extent might their CSR activity be seen to be a serious attempt to achieve SRM in the company?
- What proportion of the description of the company’s CSR activity related to external charitable donations?

The third of these sub-objectives seemed appropriate because, as will be explained in the third section (literature review), charitable donations made by listed firms are quite common and there is some literature on the motivations for such activity. As we shall see, there is some suggestion that making charitable donations is a relatively cheap and painless way in which to gain good publicity. Whereas, genuinely adopting an SRM approach is more expensive and difficult, even if it is easy to argue that morally such is the right way to behave. Before dealing with these sub-objectives we first examine the background literature which sets the scene for them.

It is worth explaining here why we focussed our attention on the ARs as the primary source of information about firms’ CSR activity. The annual report is the main feedback mechanism for communication with the majority (by number but not necessarily by proportion of shares held) of the shareholders or owners of a company (institutions with large shareholdings typically benefit from additional private briefings). It also lies in the public domain. If a firm is strongly committed to CSR, or even better to an SRM approach, then one would reasonably posit that it would be expected to make this very plain in its AR. Chan et al. (2014) also focussed on the AR as their data source in their Australian study aimed at finding whether there might be an association between declared CSR activity and corporate governance ratings.

## Research Methods

The research approach used in this paper is primarily qualitative (see e.g. Collis and Hussey, 2003): a process of logical argumentation informed by secondary data is employed. Those data came from the literature reviewed in the next section and then the contents of the sample companies’ annual reports (AR) in the fourth section which specifically addresses the three sub-objectives articulated above. The argumentative process was enriched by the use of what may be described as some soft data analysis of the data culled from the ARs of our sample. The nature of the two-part sample, the subjective scoring approach applied to the raw data and the simple statistical analysis deployed are explained below.

The data for the soft analysis were gleaned from a sample of 36 companies listed on the London Stock Exchange, all bar one from the FTSE 350 as constituted in the summer of 2019. The exception was Hornby plc, makers of train and other types of models: they are listed on AIM, the UK’s minor caps index. The empirical data from ARs came from two sub-samples: 24 companies in my own portfolio (a ‘self owned’ subset), which excluded ‘oddities’ such as gilts for obvious reasons, and a ‘control’ subset of 12 other firms, chosen to broadly match the shape of the self owned set. These data was collected in July (self-owned) and August (control) 2019.

After identifying where, if at all, reference was made to CSR issues, such references were searched for evidence of the character of any CSR activity and judgements were made of the extent to which such activity might go beyond ‘token’ activity (i.e. activity designed to offset potential criticism on the one hand and earn credit in P.R. terms on the other hand) towards a real SRM approach to management. These judgements were reflected as subjective scores on a 7-point Likert scale, see Likert (1967). An attempt was also made to assess the extent to which each firm’s CSR activity relied on charitable giving to local or inter/national charities. These judgements were again recorded as subjective scores on a 7-point Likert scale, a high score indicating heavy emphasis on charitable giving. While such charitable giving may be thought commendable in itself, it can sometimes be something of an ‘add-on’, a marginal commitment but one which gains good publicity, see the next section. The subjective scores were tabulated and some simple, descriptive statistics are given.

It is worth noting that the scoring process was consistent in that one person, namely me, did all the scoring. Hence, if there was any bias in the scoring it was a consistent bias. In an ideal world these sorts of subjective ratings would be validated by the use of a panel of competent people to replicate the scoring. In practice, getting a body of third parties to plough their way through 36 annual reports, with no obvious pay-off for them, is not realistic. The other point to note here is that the scores were based on what the companies themselves wrote in their formal documents. If any of the firms were to say that the scores do not fairly reflect their true position regarding CSR, then the reply would be that they must not have clearly reflected their activities in their published reports.

The rating process delivered two ratings or scores for each firm in the two sub-samples, the first seeking to judge how far the firm’s CSR activities, as recorded in their AR, may be thought to arrive at the end-point of genuine SRM. The second tried to assess how much of the firm’s CSR comprised charitable giving. Because we have two sub-samples we were able to compare the mean scores of the sub-samples for each of the two scoring variables, using a t-test.

The self-owned sub-sample covers a fair range of types of business (see the Appendix for more detail.) Some are highly international (such as IHG, the hotels group, and BAT, in tobacco), others are more UK centric (for example Greggs, a retail bakery chain, and Fullers, a pub and restaurant chain, the original brewing business having been recently sold off to Japanese brewers). In addition, the firms were chosen such as to offer a sufficient degree of diversification to adequately lay off risk, given my medium-range appetite for risk in my portfolio. For a discussion of the ideas underpinning investors’ share portfolio choices and their associated risk, see any, good finance text, e.g. Brealey and Myers (1996). Thus, although the sub-sample is a convenience sample, it seems fair to claim that it is not unrepresentative of the boards within the FTSE which it reflects. The control sub-set comprises firms selected to provide a set which reasonably mirrors the 24 in terms of size and scope and thereby allows some check for bias inherent in the initial sample. The overall sample is approximately a 10% sample of the FTSE 350. Furthermore, while an overall sample size of 36 is modest, it is sufficient that any statistics can be thought to have meaning from a technical standpoint.

The paper ends with a discussion which seeks to blend the underlying thinking with the empirical data, of both types, leading to pointers for desirable future actions for firms. This synthesising of ideas and findings to deliver conclusions which are actionable is a clear example of sensemaking as discussed by Weick et al. (2005).

## The Nature of CSR and Its Role

Before looking a little more closely at CSR, consider first corporate governance (CG). All firms listed on the UK’s main FTSE boards are required to include a section on CG in their annual reports. This is important in our view because, once we think what CG and CSR are about, it might seem obvious that the place to report on CSR might be under the CG section – in fact this turns out not to be the case in most of the sample firms examined, but not in all cases.

In the UK, effectively the first CG Code for the UK was produced by the Cadbury Committee in 1992, see Cadbury Report (1992). Its paragraph 2.5 is still *the* (or certainly ‘a’) classic definition of the nature of CG:

“Corporate governance is the system by which companies are directed and controlled. Boards of directors are responsible for the governance of their companies. The shareholders’ role in governance is to appoint the directors and the auditors and to satisfy themselves that an appropriate governance structure is in place. The responsibilities of the board include setting the company’s strategic aims, providing the leadership to put them into effect, supervising the management of the business and reporting to shareholders on their stewardship. The board’s actions are subject to laws, regulations and the shareholders in general meeting.”

Matters most commonly covered in the CG sections of UK company annual reports are: the Board and its effectiveness, appointment processes for board members, remuneration policy and activity, audit activity and a statement regarding compliance with the current UK CG Code, FRC (2018).

In terms of pure logic, issues pertaining to CSR might reasonably be considered to be a subset of the set of issues comprising CG. Hence one might expect to find CSR issues reported in the CG report section of listed companies’ annual reports. This is not necessarily what one finds as we shall explain in the next section.

One early, authoritative view of CSR comes from Howard R. Bowen in his book “*Social Responsibilities of a Businessman*” (1953). Bowen argued in essence that the social responsibility of the manager should go beyond profit-maximizing to meet the wider needs and values of civil society. This view establishes the need for an ethical credo for business. However, what exactly CSR should comprise has been a matter of argument, even to the extent of a lack of consensus on a universal definition and hence model for CSR activity (McWilliams et al., 2006; Meehan et al., 2006). Indeed, despite the fact that, as we stated in the Introduction, the term CSR is self explanatory, as is (too) often the case in academe, waters are muddied by writers introducing complexity and hence confusion to little real purpose.

CSR can have several aspects which include: the prudent and/or sustainable use of resources, especially those which are hard to replace; the adoption of ‘green’ manufacturing and operating procedures (e.g. non-polluting modes of manufacture, power generation and transport); and the notion that workers employed by the firm have a right to decent treatment while they labour, an idea not fully recognised until well into the twentieth century and still abused quite widely in some parts of the world. It is also notable that, while most major corporations in western economies, appear to espouse the cause of CSR, it is not infrequent that such activity seems to be something of an ‘add on’ rather than being a core driver of the business. Even when there are established units within firms, focusing on CSR, types and levels of activities vary and importantly are not always fully integrated into daily practices and strategies (Liedtka, 1998; Zadek, 2004; Kotler & Lee, 2005; Porter & Kramer, 2006). In our terms, the knee is bent somewhat to the demand for evidence of CSR but it does not amount to the embracing by the firm of genuine SRM. It should be noted, however, that this view, with which we concur, that CSR is often still a bit of an ‘add on’, is not shared by some. Moura-Leite and Padgett (2011, p. 528) concluded from their extended literature review that while CSR was something of an adjunct activity through the 1950s–1990s, “in [the] 2000s CSR became definitively an important strategic issue.” As we shall see later, this finding does not entirely resonate with our empirical data.

Nevertheless, Sirsly and Lvina (2019) suggest that matters are moving in a ‘positive’ direction. They argue that evolving stakeholder expectations are likely to push organisations to enhance their social performance [their phrase, which we suggest means ‘proper CSR’] in order to earn reputational benefits. In short companies will do good so that they are seen in a positive light. One interesting finding from their empirical work is their suggestion that the effect they posit was most strongly found in the manufacturing sector. Clearly these results are only indicative but, if well founded, they might suggest things are actually moving in a positive direction when compared with the views of McWilliams and Siegel (2000) who concluded 20 years ago that CSR had at best a neutral effect on financial performance. By contrast, Cespa and Cestone (2009) espouse a rather negative outlook on CSR. They suggest that one argument for engaging with social activist groups is that shareholders interests are better served when the protection of stakeholders is not left to the CEO’s discretion. They also assert that there is a commonly received wisdom in corporate life that CSR activities are frequently undertaken, and such action endorsed by shareholders, simply to avoid costly boycotts of their firms.

Again on the cynical side, Lim and Tsutsui (2012) found that, while global institutional pressure via non-governmental links encourages CSR adoption, such pressure ultimately gives rise to ‘ceremonial commitment’ in developed nations, by contrast with more positive reasons in developing countries. The authors developed their own framework for assessing CSR which they felt to be better suited to their projected analysis than other schemes such as the UN’s in wide circulation.

As noted by Foster (2018), while the formal notion of CSR may be a post-1950 term, some key examples may be found of businesses which have been run on an ethical, socially responsible basis well over half a century prior to the emergence than of the CSR term. Not only can one find examples of socially responsibly managed firms prior to the emergence of the formalised CSR literature after the second world war, there is also evidence of some leavening of the profit motive by moral principles in pre-twentieth Century economic writings, see Foster (2018). There remains a tension between the prevailing economic and social responsibility mantras relating to the firm. It is our view that the modal behaviour of firms is the pursuit of economic objectives centred on optimising an economic utility function, where marginal gains deliver super-normal profits (or economic rent) for shareholders, while following ethical or moral objectives less assiduously.

Lantos (2001) talks of several sub-varieties of CSR: ethical CSR – all activity should be within the law and not be in any sense morally reprehensible; strategic CSR – considerations which may not be absolutely required to make the business run but which have an immediate economic return for the owners; and altruistic CSR by which he means looking at truly philanthropic actions as part of doing business. It is the third sub-variety which if pursued vigorously might help to propel the firm along the road towards fully fledged SRM. Sadly he seeks to argue that it is not a ‘legitimate role of business’.

In the Introduction we set out three main sets of potential beneficiaries from the activities of a firm: the firm’s shareholders, its employees and its host community (be it at local or national level). To use a frequently used term these are some of the main stakeholders in a firm, along with its customers. How then do the differing stakeholders fit into the CSR pattern? We consider them in turn but in reverse order for ease of explanation.

### CSR and the Firm’s Host Community

Any firm has a host community. For a small firm this will typically be a single town or small area. For a large firm it will probably have multiple host communities, perhaps not all in one country. One of the most obvious, possible socially responsible acts the firm may make is to employ local residents and treat them well – good working conditions, attractive salaries and career development opportunities, perhaps with allied training programmes. This may seem obvious but not all firms do commit to seriously developing their staff to their full potential. Another opportunity for CSR actions is the area of waste generation and its disposal, be it atmospheric polluting emissions from production processes – CO_2_ and sulphurous emissions from power stations for example; or minimisation of waste product and packaging from shops; or the carbon based emissions from heating and cooling systems at any office block.

These are tangible aspects of the firm’s own business. Another area of CSR activity aimed at the host community is support of local educational institutions and charities. This may be simply giving money or it could be more proactive, for example affording employees the chance to volunteer with local charities for a certain number of days per annum whilst being paid their salaries. A very up to date example is Greggs (the bakery chain) working with charities to provide breakfast clubs for primary school children, thereby ensuring that no child at those clubs need start the school day hungry and hence with poor attention, Greggs Annual Report (2018).

Campbell et al. (2012, p. 99) claimed that their analysis showed, inter alia, that, “foreign affiliates from more distant home countries are less likely to engage in CSR than affiliates from more proximate home countries.” This is another version of the cynical motivation stream of thinking. [A word of caution is needed regarding this paper’s modelling. Their dependent variable in a suite of regressions was a strong CSR presence, which was represented by a dichotomous proxy. The appropriateness of such a proxy to represent what is in reality a complex, multi-faceted concept is extremely dubious. There are other aspects of their models’ structures which are less than satisfactory, so the results should be viewed with some caution.] Interestingly, one of our sample companies (PZ Cussons) made explicit claim to behave in a manner contrary to this line of thinking in respect of education for their overseas staff (see Appendix). This whole area is one where there seems to be no neat pattern across firms.

### CSR and the Firm’s Employees

The most basic corporate responsible action regarding employees is to pay adequate wages to even the lowest ranked workers and to provide a workplace environment that is pleasant and not harmful to their health. Some companies do that bit of the job tolerably well but are less mindful of the employees of their sub-contractors or suppliers. For example, how much does the seamstress sewing the cheap cotton shirt or underpants in Bangladesh, Egypt or Cambodia get paid. Moreover, is her workplace safe? Such persons may not be on ‘our’ payroll but a CSR affirming business will take steps to ensure that the answers are acceptable by UK or US standards.

The example of the Asian sweatshop is, sadly, quite widely known but other more subtle examples are to be found closer to home. Imagine you attend an event at a luxury hotel in central London say. After the talks, discussion or whatever a buffet lunch is served. How much are the waiting staff paid? You may imagine that because XY Hotels plc state in their annual report that their staff are all paid the ‘London living wage’ (LLW) - which is a couple of pounds an hour more than the UK’s national, legal minimum wage – that the answer is the LLW. You may well be wrong because some or most of the waiters on parade may be agency staff and the agency pays them the national minimum wage. This may not be the near penury suffered by the Asian seamstress but it leaves little if any slack in the worker’s budget. This example is not imaginary: I have personally checked the story.

Cadbury’s famously built their model town at Bournville near Birmingham, see e.g. Morland (2009). They also gave educational opportunities to workers: all this in the nineteenth century! Some firms set up their own in-house colleges, and in a few cases even universities (McDonalds in the USA for example), to afford staff the chance to improve themselves at the employer’s expense.

### CSR and the Shareholders

The issue regarding shareholders and CSR can be seen from two conflicting perspectives. On the one hand there is the Friedmanite view that companies have no duty other than to maximise the wealth of their members (/shareholders), Friedman (1962, p. 133). On the other hand there is the view that decent citizens who happen to be shareholders should reasonably wish that the companies in which they own shares should, like them as individuals, also act decently, morally, ethically. The latter view might mean that, if one seeks to keep down costs of manufacturing by producing in third world countries, one still has ethical duties. One should at least do the following: only employ adult workers; pay those workers decent wages in PPP terms; provide a safe and healthy working environment. One might also go further and add healthcare benefits, subsidised housing and school support for their workers’ children. To act in the manner described seems a reasonable ethical or moral stance to adopt but does everyone meet those aspirations? No is the short answer. Finally in this subsection, we note that there is a perfectly good argument to the effect that not only is such activity the decent way to behave but it also is in the interest of shareholders ultimately. In other words by behaving ethically the firm does better in the long run, see e.g. Carroll and Shabana (2010). As the cynic might have it, it is good advertising copy to be seen to be ethical, to do good.

However, Ben-Amar and Belgacem (2018) suggest that companies may act primarily from cynical motives, by massaging perceptions of their firms by the way in which they report CSR activity. Based on a sample of large firms listed on the Toronto Stock Exchange, their results were said to show a positive association between corporate social performance and the syntactic complexity of the management’s discussion and analysis section of the annual report. They interpret these findings as suggesting that managers may engage in CSR opportunistically and use complex narrative disclosures in a somewhat cynical attempt to manage impressions of the firm.

We have focussed on the three highlighted areas because they are ones which do feature regularly in firms’ statements of CSR activity. The other matter we have perhaps overlooked thus far but which needs attention is the need for socially responsible businesses to operate and trade in an *ethical* manner at all times, to engage in ‘ethical CSR’ as Lantos (2001) would have it. Obvious perhaps but is it always so? One example where the otherwise ‘blameless’ firm may fall below the highest standards is in the matter of paying taxes. Especially for internationally focussed firms there are many perfectly legal devices for minimising corporate tax bills. They are legal because the tax codes of the UK, the USA, France or wherever are framed in such a fashion that avoidance schemes used do not break the law. If, however, one asks whether it is ethical to use off-shore intermediary entities based in tax havens to avoid corporation tax in the country/ies where the profits are really earned then the answer may be less favourable to the firms in question. However some firms do claim that they make real efforts to pay fully taxes on what they earn where they earn it, for example Informa, one of our sample of firms (see Appendix.)

Nevertheless, even if tax avoidance schemes are somewhat lacking in ethical terms, they are not illegal. What is illegal in almost any country in the world is bribery – large or small – but it remains prevalent in many countries. There is also the problem of even intelligent, ostensibly decent folk saying that they see the distinction between big bribery and small-scale sweeteners or gift giving. Right now Malaysia has a big corruption scandal around the government’s 1MDB fund. Everyone to whom I have spoken in Malaysia sees that as a problem but some intelligent young people in an MBA class I addressed in 2017 tried to argue that, by contrast, ‘gift giving’ is not really corrupt. I demurred. Both the UK and the USA have laws dealing with off-shore (to them) bribery, Bribery Act, UK (2010) and FCPA, USA (1977, 1998), but there are still cases in which UK and US firms get caught up. It takes two to tango, as the saying goes. In other words, for such corrupt payments to occur there have to be willing recipients in the overseas country. Those parties may in fact be the initiators of the whole thing: ‘if you want to do a deal I want a cut.’

## Soft Empirical Data and Its Evaluation

In this section we shall enumerate what we found on the subject of CSR in the annual reports of our 36 sample companies. One of the things we might expect is that companies might give money to charity essentially because it is a relatively painless way of being seen to do good but does not necessarily involve the hard effort of embedding social policies into the very fabric of the firm. We attempted to search for the answers to our three research questions, beyond checking that every annual report (AR) had a section designated to reporting CG issues, all 36 did comply with this last point basically because they are obliged by a regulator so to do. As noted earlier, these three questions or issues were:

- Where within the AR did the company report on CSR activity?
- What did they report themselves to be doing on the CSR front and to what extent might their CSR activity be seen to be a serious attempt to achieve SRM in the company?
- What proportion of the description of the company’s CSR activity related to external charitable donations?

The answer to the first question was clear. In the great majority of cases there was a section of the AR named “strategic report”: this was the locus of report for CSR activity. In a few cases there was a dedicated section for CSR or sustainability issues (e.g. Informa, from the self-owned subset, and Brewin Dolphin, from the control subset) or an additional report separate from the AR (e.g. Associated British Foods, from the control subset.)

With respect to the second and third questions, our conjecture was that scores on the second question would be relatively modest and those for the third question would be higher. These scores are shown in columns 2 and 3 of Table 1a and b below. It is worth re-emphasising that, as noted earlier, the scores were based on what the companies themselves wrote in their formal documents.

**Table 1:** Assessing CSR against SRM and charity (bold entries are FTSE 100)

Company (latest AR at July 2019)	CSR ~ SRM (x_1_)	% CSR ~ charity (x_**2**_)
	Score (/7)	Score (/7)
1a – Own shares subset		
Barclays	2	3
Barr(AG)	1.5	5
BAT	2	1
Burberry	2	3.5
Fidelity Asian Values	1	1
Fidelity Sp Value	1	1
Fuller, Smith & Turner	2	4.5
Greene King*	3	4.5
Greggs (Bakery)	3	5
Hornby	1	1
IHG	5	5
Informa (post UBM)	4.5	5
Kingfisher	2	1
Land Securities	3.5	3.5
Lloyds	2	6
Marston	4.5	5
Morrison	3	5
National Grid	3	5
PZ Cussons	3	2.5
Rentokil	3	3
Stagecoach	3	5
Tate & Lyle	3	4.4
TP-ICAP	2	5
Wetherspoon, JD	2	2.5
Average score	2.58	3.64
* Greene King wholly acquired by Li Ka Shing late 2019		
1b *– Control subset*		
Assoc. British Foods	3	5
Brewin Dolphin	2.5	6
British Land	2.5	3
Britvic	2	4.5
T Clarke	3	3
Drax	2	2.5
Dunelm	2	5
Halma	3	2
HSBC	1.5	1
Imperial Group	2	1.5
Mitchells and Butler	4	2.5
Sainsbury	3	5.5
Average score	2.54	3.46

What the scores in Table 1 seem to say are this. First, while the companies do undertake CSR activities, such work is well short of achieving fully fledged SRM (the means for the sub-samples of column 2 of 2.58 and 2.54 are well below the scale mid-point of 4). Second, referring to column 3, a non-trivial element of these companies’ CSR work relates to supporting charities in the various places in which they operate (18 of the 36 scores were above the scale mid-point). This is in line with our prior predictions.

In the third column, for own shares, three companies were rated as ‘1’: two were Investment Trusts which may explain why they reported no obvious activity. The third company was Hornby which are a small, listed company and frankly speaking probably feel they have enough on their hands simply working to survive after a number of years of reported losses and problems with their supply chain.

Having two sub-samples also enables us to make comparisons between them. Comparing the sample means answers the question whether there was something unusual about the character of the own shares sample; put another way, was there some bias intrinsic to my initial share choices? Observationally the means for the control sample are not very different from those from the own shares sample. This is confirmed by conducting a t-test of the differences between the sub-sample means. Table 2 shows the means for the two sub-samples, the associated standard deviations and the value of the t-test statistic for comparing the means assuming equality of the means for x_i_ at the 0.25 level, where i = 1,2. We refer to testing at the 25% level because this illustrates just how far the alternate hypothesis is from being indicated, or, if you prefer, how strongly indicated is acceptance of the null hypothesis. Since our sample is a convenience sample, we do not presume to say that we can infer this conclusion to be true across the FTSE; rather we say it is evidence from which we may reasonably *conjecture* that such may be the case.

**Table 2:** Summary of sub-sample statistics

Sample/mean (SD)	Var (x_1_)	Var (x_2_)
Self-owned	2.58 (1.080)	3.64 (1.649)
Control	2.54 (0.689)	3.46 (1.671)
t-statistics for comparison of sub-sample means:		
t_24 + 12–2_ (x_1_) = 0.117 = › accept null hypothesis		
t_24 + 12–2_ (x_2_) = 0.307 = › accept null hypothesis		

Further data culled from the self-owned companies’ annual reports, and on which the scores were at least partly based, are laid out in the Appendix. The table gives brief details not only of where the companies talked about CSR in their annual reports, but also what they said, or claimed, that they did which could be seen as CSR activity and what their charity oriented actions, as part of the wider set of activities, were.

Careful review of these soft data show at least four things, the first three being answers to our earlier questions, as follow:

- Very few of the sample had a separate part of their annual report devoted solely to CSR issues. The most common home for such discourse was the strategic report (which typically embraces statements from the Chairman and the CEO), not the section on CG, which might be thought to be an appropriate location. What this tends to suggest is that firms reporting in the UK see CG data as a rather separate and dry set of information required of them by the listing and regulatory authorities: a task to be completed but not a place in which to show imagination or initiative.
- Our judgement, and one stresses it is no more than that, based on the data we reviewed, is that none of the sample companies had made the forward leap to being a truly SRM company, with SRM principles built into the firm’s foundations and informing *all* of the firm’s activities onwards.
- Nearly all the sample of companies donated monies to charity from their own earnings or facilitated the collection of monies from customers for onward giving to charities, for example helped collect money for charities by giving space on their pubs’ bar counters for ‘boxes’. This is of course all to the good and shows a responsible, charitable view aimed to help the less fortunate in society. The data summarised in column 3 of Table 1 tend to support the view that the companies surveyed may in some sense be taking the easy way out. They make or facilitate charitable donations. Such donations are morally approvable, they earn credit but they remain part of a semi-detached, or “add-on” narrative. The pattern is similar for both sub-samples. This is interesting in that, just over a decade ago, Brammer and Pavelin (2005) reported that UK firms performed less strongly than their US counterparts in this area.

The fourth point is this. Some firms showed interesting and arguably innovative actions within the set essayed. For example the policy of Greene King and Land Securities to try to offer jobs to ex-convicts is truly in the best spirit of CSR, Land Securities even offering a training programme specifically aimed to help such people become ‘aerial window cleaners’ (men or women in a gondola suspended down the side of a skyscraper to enable them to clean the windows or, in some cases more accurately, glass walls.) Many people in our modern society are not prepared to give those convicted of a crime a second chance. One wonders how they would feel if they were to become guests of Her Majesty’s Prisons at some point: would they become more understanding of others’ moral failures? Greggs meanwhile underwrote breakfast clubs for primary school children – in the schools served, no child would start the day cold or hungry. It is perhaps surprising that such a possibility of need might exist at all in twenty-first century Britain but having access to money does not necessarily mean that those parents who hold it will use it wisely and morally. If you have a child what could be more important than to feed or clothe that young innocent?

In an environment in which companies may now perceive a need to be seen to be ‘socially responsible’ in some way, giving to charities could be argued to be an easy way out. The sums given may sound substantial in a vacuum, or relative to our own personal budgets, but as a proportion of total earnings of decent sized companies they are usually not too onerous. Hence one takes a morally approvable action (make the donation/s) at fairly low cost which delivers pay off in terms of soft advertising about one’s firm being ‘good chaps’ (this harks back to the point referred to earlier which was made by Sirsly and Lvina (2019)). How much harder, and probably costly, it would be to make only socially responsible decisions at all times.

Making sure that the farmer is decently paid for his crop somewhere in Africa or South America or that the factory workers in a S.E. Asian sewing workshop are paid a truly living wage to work in healthy surroundings will raise costs in the supermarket or clothing retailer’s supply chain. But, if SRM is really the name of the game, that is exactly what we all must do. Moreover, when we as individuals in privileged western countries go shopping, we must not be too greedy for cheap goods.

As I was drafting this paper, the Covid-19 crisis stuck. There have been clear examples of companies large and small engaging in activities which are evidently socially responsible. These range from big firms such as Rolls Royce answering the UK Government’s request to manufacture ventilators, to local restaurants preparing food for health-care workers and others. Meanwhile, big pharmaceutical companies are engaging separately or in consortia (in some cases linking with university departments) to try to discover an effective vaccine for the new virus: if they succeed they will truly be serving their communities and those of other countries. It is both interesting and heart warming to see firms so engaging. The interesting question, from the point of view of this paper, is whether this pattern of socially responsible activity will endure when, as we hope will be the case, the crisis is over.

## Conclusion

The task we set ourselves at the beginning of this paper was to try to discover whether, in the world of the listed firm, in the UK and by extension elsewhere, there is publicly recorded evidence of CSR activity by such firms and to what extent, if any, such activities may amount to genuine SRM by the firms. We found that most companies in a sample of 36 did show some evidence of awareness of CSR issues but our conclusion, based on information in their annual reports, was that this CSR awareness and consequent activity fell a good way short of our being able to class them to be SRM firms. Such a conclusion is not meant to be particularly one of opprobrium: our conjecture is that the sample firms are probably no better and no worse in this field than many others. Moreover, to have once been something of a beacon in the territory is not a guarantee of continued moral excellence. We noted elsewhere, Foster (2018), that while Cadburys had once been a name of repute in the CSR/SRM arena, it seemed to have lost some of its lustre after being taken over by the US firm Kraft Foods in 2009. Indeed Kraft took the moral low ground in very short order. When bidding for Cadburys they promised that there would be no big job losses in Britain if they won control. In less than a 12 month they had reneged on that undertaking and closed a plant employing several hundred people.

As we noted at the outset, it is possible to run a profitable business in an SRM fashion. If a firm is closely held, especially if by a family group, then it may well be easier to aspire to operate on an SRM basis. Quite simply, the close holding group can agree among itself to use a dual faceted utility function as its guiding light where one facet is one of wealth generation and the other is one of moral conduct in all aspects of the firm’s operation (the SRM facet). In widely held companies, adopting the latter facet may be much more problematic. People ‘out there in the market’ may take exception to some element of profit being foregone in order to be socially responsible. If such investors take it upon themselves to trade the stock down in the market, life may become much harder and a high quality management team may be pushed out not because they are poor managers but because they have moral standards which others regard as too demanding. Such is the sad reality.

We know from first-hand experience that such threats are not figments of the imagination. An investment trust in whose holding company we held shares was ‘attacked’ by an American ‘activist’. Within a year, having built a big holding he (or his chosen operating vehicle) had managed to press the fund managers to strip out many of the choicest investments and sold them for large sums. In our opinion, these actions destroyed a good company, making a lot of money for the raider and leaving small investors such as us with large income tax bills caused by the unwanted special dividends which were paid as sales went on apace. Initially the raider alleged that the then board lacked competence or an effective investment strategy. He, it was claimed, had a better investment strategy to offer but strangely this new positive (presumably) investment strategy was never revealed to us mere owners of the company.

In conclusion it is a matter for rejoicing that CSR issues are at least on the corporate agenda; engagement is perhaps still a tad tentative but this is nevertheless significant. There is, however, still a long way to go before anything approaching the SRM company becomes the norm. Covid-19 has given us all cause to pause and reflect; will it be a catalyst which may help to make the SRM company a more likely presence in post 2020 society? We shall see.

Thinking about the significance of the area of work, we have made an underlying assumption that engagement in CSR and by extension SRM is a desirable trait because we feel that to be an evidently moral stance, supported to some extent by our own study. The importance of our study is, in one sense, exemplified by the fact that it is one of very few which seek to look holistically at the notion of the socially responsibly managed firm, as was noted by one of the paper’s reviewers. Hence, further, more comprehensive studies along the same lines as this one would be welcome – maybe it would suit several PhD students in different countries. But as noted earlier not everyone necessarily agrees, which we find disappointing.

Another linked question, which may be of interest to be explored, is why CSR is apparently not seen as intrinsically part of CG, as they are reported in annual reports. Is it simply that ‘the dead hand of the grey-suited accountant’ has descended on CG? For example, in the UK, the Financial Reporting Council holds the reins of the CG Code which applies to listed firms, see FRC (2018). Thus to explore this proposition would require those very grey-suited people and their regulator to agree to talk frankly and openly.

## Appendix

**Table 3:** Data sheet of CSR/SRM information culled from annual reports of “own shares” subset (most recent as at July 2019)

Name of Company	Location of CSR discourse in AR	Description and does CSR activity ~ SRM (score/7)	Degree of CSR focussed on External Charities (score/7)
Barr (AG)	Sustainability report in Strategy Report [SR] (energy, water, responsible re sugary prods)	Not really, so score 1 to 2. Co’s product responsibility is basically pressure on Industry generally about sugar in soft drinks	Mentions £150 k donations over 3 years to Macmillan cancer; support/pay employee volunteering. Score – 5
Barclays Bank	Primarily in the SR, incl. section labelled ‘Corporate Compliance’ (incl. items such as Board diversity, human rights protection, anti-bribery/corruption as stated objective)	‘Responsible’ finance for energy; Green finance for low carbon business. Connect to Work programme helping many. Imply ‘responsible banking’ too. Score 2 (possibly a bit more?)	Support for staff volunteering, Could not find overt statement of money given to charity/spent on outreach programmes as in last column. Score – 3 (taking account of said programmes)
BAT	Strategic report; includes sustainable agriculture in supply chain, support farmers, human rights	Not really: score 2	Not visible – score 1
Burberry	Mainly in SR; helping youth in supply areas, sustainable packaging, reduce water use	Only slightly: score 2	Estimate score 3.5–1% pre-tax π to social/ community causes (p105)
Fidelity Asian Values	As Fid SpV below but in SR does say they support positive ESG (environmental, social and governance) attitudes in invested entities	Score 1	Not visible – score 1
Fidelity Special Values	Nothing found really – assume since special Co type	Score 1	Not visible – score 1
Fuller, Smith & Turner	There is a report in front section, labelled CSR. Claim: aim to be good employer; eco-friendly sourcing; low polluting etc. Surely this is just basic in 2019, makes a story though!	Not to great degree – score 2	Fair bit – score 4.5. Over £150 k to charity or >0.75% pre tax π (which was £20.9 m)
Greene King (acquired by Li Ka Shing vehicle in late 2019 – so no longer quoted on LSE)	Soc. Resp. policy in SR: says put people 1st; support local communities; resp. retailing & operate sustainably	In their SR, exx include: reduce sugar in drinks/food; compostable straws; employ ex-cons; Greene Kingdom programme includes learning modules on work and personal issues. Score 3	Score 4.5. Raised £1.4 m via collection boxes on bars for Macmillan cf. pre-tax π £245 m
Greggs	Nothing with said heading, but see comment in next column	In CEO’s report, mention ‘making Greggs a good place to work’, minimise waste, Board has 4 male/3 female (or 43% female which is high vs. most listed firms). Aim to source product responsibly. Score 3	Policy to give min 1% pre tax π p.a. to Gregg’s Foundation. GF funds local community projects incl. Breakfast Clubs via 80 partners for primary school children. Score 5
Hornby	N.A. - in CG Report, new to AR for 2019, state aim to be ethical in their trading)	No – score 1	Not visible – score 1
IHG	In CG Report mentions CR Cttee. SR (staff incl. IHG Academy but pay in UK??), suppliers, reduce environmental impact, local charities - IHG Foundation)	Quite good around staff etc. in developmental terms, including overseas. Recent announcement- no individual tubes/bottles of bathcare - Pay issue is probably one of IHG employees vs. franchisees’ staff. Score 5 (?)	Score 5
Informa	Separate Sustainability Report: doing regular business ‘greenly’, minimise pollution, support (paid leave) volunteering	Overall they do seem to report a real effort (or they are v. good at storytelling), so score 4/5 (?) One thing is an affirmation to fully pay taxes wherever they work	Quite heavily so, score 5
Kingfisher	In CEO’s report. They have a group sustainability committee. Basic work re production & products, hence customers’ homes	Not a lot, score 2	Score1 –not visible
Land Securities	SR: Social approach in Strat. Review, pp. 45–53	Claim (implied) social value contribution of community programmes estimated at £3.2 m. They try to help ex-cons and perhaps while still cons (e.g. ‘aerial’ window cleaning prog.); educational outreach to kids; charity partnerships – ‘donation’ can be making space available Score 3/4	Score 3.5
Lloyds Bank	In SR; in CG report there is also a brief statement of ‘what we did’ vis a vis social responsibility actions	They use 4 ‘Independent Charitable Foundations’ (ICF) to funnel support to over 3000 bodies. In house, they, like many, comment on inclusivity, equality, human rights etc. (but not really SRM, just basic good mgt.) Score 2	We are one of the UK’s largest corporate donors’, incl. to/via its ICFs. On p22 in SR noted £100 m charitable donations in 2018, approx 0.6% of net income. Score – 6
Marstons	SR- section on CR. (w.r.t. staff, suppliers, cutting environmental impact, local charities)	To a degree – score 4/5 (?)	Quite a lot, score 5
Morrison Wm.	P21, CR in SR (there exists Corporate Compliance and Resp. Cttee; refer to stuff like Health & Safety, ethical trade, reduce waste and/or pollution)	Good employer (in SR), M do talks etc. in schools, hope to get future staff thereby. Score – 3	Over £3 m to charities incl. Cancer (CLIC Sargent - child cancers) - approx 1% pre tax π- score 5
National Grid	Have Envt/Health Committee; plan to add CSR session to regular Board roster	Claim embedding sustainability into strategy. Aim to reduce greenhouse gas emissions; manage waste more effectively; caring for natural envt. (think – they have big items on the hillside). Promoting skill sharing via volunteering →STEM subjects	In UK help Affordable Warmth Solns CIC, put £49 m to Warm Homes Fund; put 1st time CH into ‘vulnerable homes’; $3 m p.a. → Charities in US? Score 5
PZ Cussons	Such as there was in SR; Community & Charity heading in Good4Bus section	Aim to operate ethically; values as enablers; apprentices/internships/Grad Training, especially in Nigeria and Indonesia~ bonus pts.; separate doc on CSR policies-Modern slavery, Group Tax Strategy and sourcing Palm Oil well. Similar to Morrisons? So score 3	Not really clear in docs but most firms do so ‘guess’ score – 2.5
Rentokil	SR; Usual stuff on running business “well” re, employees, supply chain, minimise CO_2_ etc.	Re communities spoke of Charity giving specifically. Refer to women on Board, diversity & in-house training via on-line units at U+. Score 3 perhaps	Record spend of £202 k, .08% of Op π, not incl. benefit in kind, score 3-?
Stagecoach	In SR (pp1–29) says ‘Do Business in SR(M) way’: s.1.8.1, provide socially useful transport; employee training etc.; reduce pollution e.g. adopting electric vehicles	Not a lot on CSR per se, bar SR(M) actions in previous cell, so score 3 (?)	Charity money seems a lot if right at c.5% of pre-tax π – score 5
Tate & Lyle	Pp 56–57 in SR (Community Involvement programme ~ Health, Hunger, Education: overall strategic objective to reduce sugars in food)	They ‘try’ to a degree, score 3 (?)	Quite a lot (£500 k spent, 0.02% vs pre tax) but cf. Stagecoach. Score 4.4
TP-ICAP	In CG report there is mention of CSR strategy (talks of its development and delivery; developing ‘our people’; continue to run the ICAP Charity Day – £4.5 m)	Generic good employer stuff -score 2	Charity money to multiple charities around world (~2% pre tax π) Score 5
Wetherspoon J.D.	Implicit in SR (Protect Human Rights; training staff; monitor food hygiene. Bonus % for staff from π), separate short doc on Community and Charity	Just as decent employer-score 2	Charity includes British Legion poppy collection c. £25 k p.a.. Also RNLI, some and local charities (cf. £107 m pre-tax π.) Score 2.5
